# Antimicrobial use and resistance profiles of *Escherichia coli* and *Campylobacter* species from small-scale poultry farms in Central Kenya

**DOI:** 10.1186/s12917-026-05505-9

**Published:** 2026-04-27

**Authors:** Christine M. Mbindyo, Luciano A. Achieng, Daniel W. Wanja, Shepelo Getrude Peter, Felix M. Kibegwa, Romona Ndanyi, George C. Gitao, Charles D. Kato

**Affiliations:** 1 Department of Veterinary Pathology, Microbiology and Parasitology, Faculty of Veterinary Medicine, University of Nairobi, P.O. Box 29053- 00625, Nairobi, Kenya; 2 Department of Veterinary Pathology, Microbiology and Parasitology, Faculty of Veterinary Medicine and Surgery, Egerton University, P.O. Box 536-20115, Njoro-Egerton, Kenya; 3 Department of Clinical Studies, Faculty of Veterinary Medicine, University of Nairobi, P.O. Box 29053-00625, Nairobi, Kenya; 4 Department of Animal Production, Faculty of Veterinary Medicine, University of Nairobi, P.O. Box 29053-00625, Nairobi, Kenya; 5 Directorate of Veterinary Services, Ministry of Agriculture and Livestock Development, P.O. Box private bag-00625, Nairobi, Kenya; 6 School of Biosecurity, Biotechnical and Laboratory Sciences, College of Veterinary Medicine, Animal Resources and Biosecurity, Makerere University, P. O. Box 7072, Kampala, Uganda

**Keywords:** *Escherichia coli*, *Campylobacter coli* and *C*. *jejuni*, Antimicrobial Resistance, Poultry, Smallholder farming, Kenya, Public health

## Abstract

**Background:**

Globally, *Escherichia coli* (*E*. *coli*) and *Campylobacter* spp. are key foodborne pathogens and important indicators for antimicrobial resistance (AMR) surveillance in poultry. Our study assessed their occurrence, antimicrobial resistance profiles, and antimicrobial use in Kenyan small-scale poultry production. A cross-sectional study was conducted between August and December 2024 in 340 small-scale poultry farms across four sub-counties of Murang’a County. In each farm, cloacal swabs from five adult chickens were aseptically collected and pooled to form a composite sample, while data on antimicrobial use were collected using a semi-structured questionnaire. Isolation of *E*. *coli* and *Campylobacter* spp. was carried out using conventional culture procedures, and isolates were confirmed with matrix-assisted laser desorption/ionization time-of-flight mass spectrometry (MALDI-TOF). Antimicrobial susceptibility testing was performed using the Kirby–Bauer disk diffusion method, as per the CLSI (2024) guidelines.

**Results:**

Antimicrobial use was reported in 75.3% (*n* = 256/340) of the study farms, with tetracyclines 73.8% (*n* = 189/256) and sulfonamides 52.3% (*n* = 134/256) being the most commonly used classes. *E*. *coli* was detected in 99.4% (*n* = 338/340) and *Campylobacter* spp. in 61.4% (*n* = 209/340). Among the *Campylobacter* isolates, *C*. *jejuni* 58.8% (*n* = 123/209) was more prevalent than *C*. *coli* 41.1% (*n* = 86/209). The highest resistance among *E*. *coli* isolates was observed against tetracycline, 74.0% (*n* = 77/104), and sulfamethoxazole-trimethoprim, 65.4% (*n* = 68/104). Extended-spectrum β-lactamase (ESBL)-producing *E*. *coli* and phenotypic carbapenem-resistant *E*. *coli* were detected at 4.8% (*n* = 5/104) and 25.0% (*n* = 26/104), respectively. *Campylobacter* spp. exhibited the highest resistance to nalidixic acid 67.3% (*n* = 69/104) and tetracycline 65.4% (*n* = 68/104). Multidrug resistance (MDR) was identified in 58.6% (*n* = 61/104) of *E*. *coli* and 46.1% (*n* = 48/104) of *Campylobacter* spp. A significant correlation between antimicrobial use and *E*. *coli* resistance profiles was found in Kigumo sub-county (*r* = 0.72, *p* = 0.04), whereas no significant association was observed for *Campylobacter*.

**Conclusion:**

Extensive use of antimicrobials and high multidrug resistance among *E*. *coli* and *Campylobacter* spp. in small-scale poultry farms in Kenya pose a significant public health and food safety risk. Therefore, targeted strategies to reduce antimicrobial use and the emergence of AMR bacteria from poultry production are required in the region.

**Supplementary Information:**

The online version contains supplementary material available at 10.1186/s12917-026-05505-9.

## Introduction

*Escherichia coli* pathotypes and *Campylobacter* species are among the leading causes of enteric infections in humans worldwide and in Africa, resulting in millions of deaths [1–4]. It is estimated that almost 1 in 10 people get various foodborne infections from contaminated foods, and 420,000 people die every year worldwide [1]. These infections are commonly caused by major bacterial pathogens such as *Salmonella* spp., *E. coli*, *Campylobacter* spp., and *Listeria monocytogenes* [2, 3]. Chickens and their products are some of the main reservoirs of these foodborne pathogens and constitute major pathways through which humans become infected [5, 6]. Pathogenic *E*. *coli* pathotypes may cause systemic or localized colibacillosis in birds, while commensal strains form part of the normal gut microbiota and may contribute to colonization resistance [7]. Human pathogenic species *Campylobacter jejuni* and *C*. *coli* frequently colonize the gastrointestinal tract of chickens and are mostly asymptomatic [8, 9]. However, these pathogens pose a significant public health threat to humans, causing infections such as enteritis, bloodstream infections, and urinary tract infections, while also serving as reservoirs for antimicrobial resistance (AMR) [7, 10].

WHO 2015 describes the consequences of unaddressed AMR as high human deaths, huge economic losses, and food insecurities [1]. The highest burdens are expected in lower resource settings like rural communities of Africa and Kenya [11–13]. These regions have low healthcare infrastructure, sanitation, and generally have intrinsic livestock-human relationships [13, 14] which allow for cross-infections of these AMR bacteria across animal species and humans [15, 16]. Practices such as unrestricted access and widespread use of antimicrobials in poultry production are common practices in Kenya and key contributors to the emergence and spread of AMR bacteria [17, 18]. Further, AMR bacteria can be transmitted to humans via the food chain and persist in the environment [16, 19].

In Kenya, over 80% of the poultry production system is small-scale, with the majority of chicken reared being indigenous (local/natives) and improved (crossbred) breeds [18, 20, 21]. These chickens are raised for commercial and local consumption under poor farm biosecurity measures and a high disease burden, likely driving antimicrobial misuse/overuse at the farms [20, 21]. Indeed, over 86% of poultry farmers in Kenya have been reported to self-administer antimicrobials without proper regulation or veterinary supervision [18, 20]. This unregulated use of antimicrobials is likely accelerating the development of AMR and MDR in foodborne bacteria, including *E*. *coli* and *Campylobacter* spp. in chicken, posing a major public threat to human health [5, 14].

Globally, in recent years, bacterial foodborne pathogen resistance to antimicrobials has gradually increased [1, 2]. In addition, the prevalence of MDR strains has also increased, making foodborne infections from these pathogens difficult to treat, including in Kenya [2, 5, 11]. In Central Kenya, indigenous and improved chicken farming is an emerging significant agricultural activity for nutrition and income of households. Given this context, currently there are very limited studies on the occurrence of foodborne bacteria and their antimicrobial susceptibility profiles, especially *Campylobacter* spp. from chicken at the farm level in Kenya, including central Kenya [14, 22, 23]. Furthermore, current evidence on AMR in foodborne bacteria in Kenya is largely derived from studies on exotic chicken breeds in urban and peri-urban areas, with a significant knowledge gap in indigenous and mixed-breed chickens in rural production systems [22–26]. Therefore, the current study investigated the antimicrobial usage and resistance of *E*. *coli* and *Campylobacter* species isolated from chickens from small-scale poultry farms in central Kenya.

## Materials and methods

### Study area

**Fig. 1:**
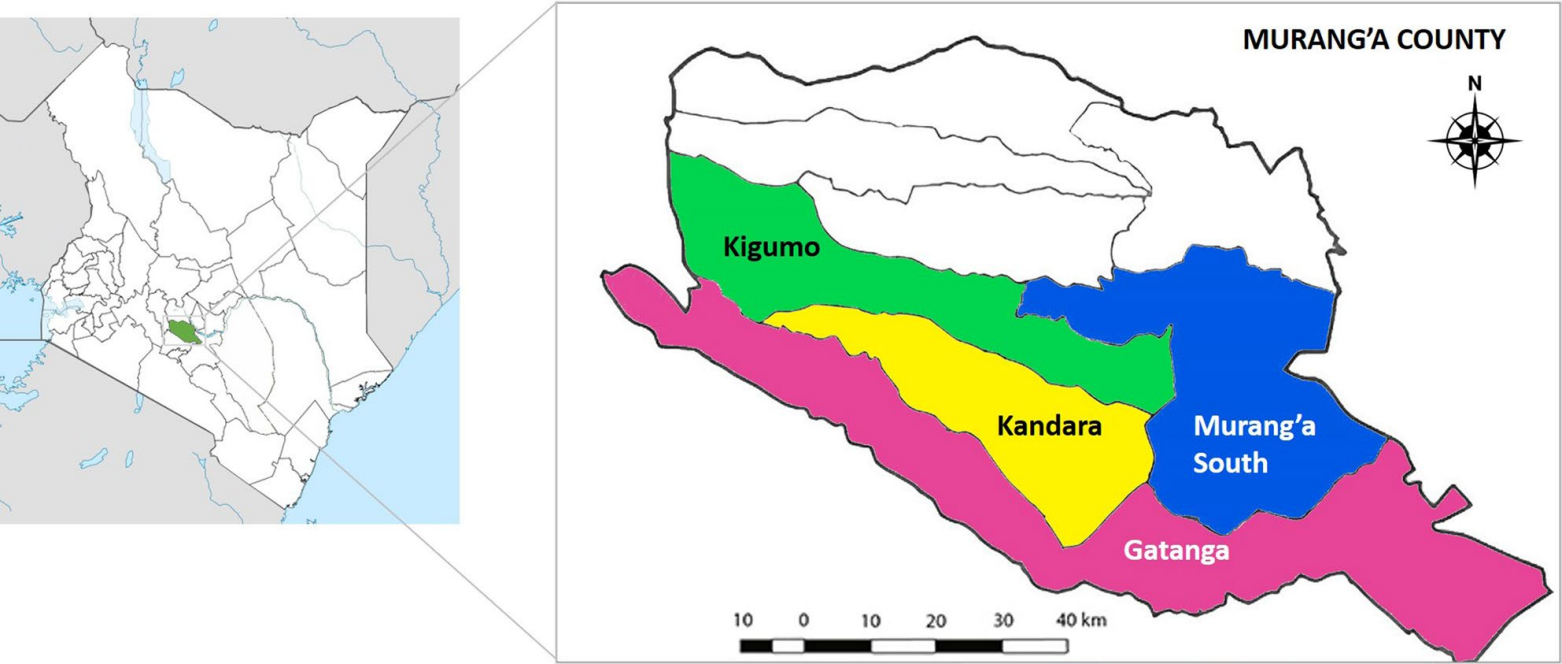
Map of Kenya showing the location of Murang’a County and the specific sampling sites within the county

The present study was carried out between August and December 2024 in four sub-counties of Murang’a County, Kenya, namely Murang’a South, Kandara, Gatang’a, and Kigumo. Murang’a County is situated in central Kenya, approximately 84 km north of Nairobi (Fig. 1), which lies between latitudes 0°43’0’’S to 1°10’0’’S and longitudes 36°48’0’’E to 37°10’0’’E. According to the Kenya National Bureau of Statistics (KNBS) [27], Murang’a County contributes 2.3% of the national Gross Domestic Product (GDP) and is a major agricultural county in Kenya with well-established smallholder mixed-livestock production systems with high numbers of poultry [27]. Murang’a County is known for its fertile lands and favourable climates, which are ideal for diverse agricultural activities, including tea, coffee, dairy, and poultry farming. Due to the small nature of land sizes in the region, there is a close relationship between humans and animals. These sub-counties were chosen for their extensive chicken production systems and accessibility [28].

### Study design

This cross-sectional study was conducted in small-scale poultry farms in Murang’a County, where pooled cloacal swabs from chickens were collected and analysed for bacterial carriage and antimicrobial resistance among the isolates. Local/indigenous chickens (local native breeds), improved /mixed breeds (genetically enhanced or crossbred chicken strains), broilers and layers, under either intensive, semi-intensive, or free-range production systems, were sampled. The small-scale poultry farms here were characterized by farmers owning at least 10–500 birds. Small-scale farms, also referred to as sector 3, are commercial poultry production systems with low to minimal biosecurity and birds/products entering live bird markets [21]. Farms not keeping chickens and or large-scale as well as those that declined to participate were excluded from the study.

### Sample size estimation and sampling strategy

Small-scale farms were the sampling units in the study, and they were enrolled based on the following criteria: (1) actively engaged in small-scale chicken farming with a flock size of at least 10 and a maximum of 500 for the past 1 year. The sample size was estimated using a formula described by Martin et al. [29]; $\:n=\frac{{\mathrm{Z}{\upalpha\:}}^{2}pq}{{L}^{2}}$. Where ‘n’ was the required sample size, ‘Z_α’_ was 1.96, the standard normal deviate at 5% level of significance, ‘p’ was the estimated prevalence at 0.5, while ‘q’ was ‘1-p’, and ‘L’ was the precision of the estimate and was set at 5%. In the absence of prior prevalence estimates for the study population, a 50% assumed prevalence was used in the sample size calculation to provide a conservative estimate that yields the maximum sample size. The required sample size, n, was 384 farms. The farms that fitted the above criteria were randomly selected within the strata from a list obtained from the County Director of Veterinary Services office and included in the study. However, in our study, only 340 farms were sampled based on accessibility and willingness to participate. In each farm, a representative sample of 5 different adult chickens was randomly selected and, after restraining, sampling was done according to the standard guidelines.

### Questionnaire administration

At each farm, a pre-tested semi-structured questionnaire was administered to the owner of the chicken farm to collect data on antimicrobial usage and other farm biodata. The questionnaire was initially pretested to assess clarity and completion time, and subsequently validated. On the day of the farm visit, the research team explained the project objectives to the farm owners of the selected households, and informed consent was obtained from the individual participants. Formal structured interviews were then conducted using a questionnaire designed in Google Forms. The survey captured data on types of antimicrobials used, frequency and purpose of use (therapeutic, prophylactic, or growth promotion), sources of the drugs, and other farm management characteristics relevant to antimicrobial use. As further evidence of antimicrobial use, farmers were requested to show any available drug sachets, containers, and drug bins (containers used for disposal of antimicrobial products) present at the farm. All the data were anonymized to ensure confidentiality and protect participants’ privacy. The original questionnaire is provided online in the supplementary section as Supplementary File S1.

### Sample collection and transportation

A total of 340 cloacal samples were collected aseptically from chickens using sterile swabs and immediately placed in Amies transport medium. On each farm, five chickens were randomly selected and sampled, with duplicate cloacal swabs collected from each bird. One swab from each bird was used for the isolation of *Campylobacter* spp., while the second swab was used for the isolation of *E*. *coli*. The five birds sampled per farm were considered representative of the farm, and together constituted one composite farm sample, resulting in 340 farm-level samples included in the study. The reason for this approach was to account for within-flock variability in bacterial carriage and improve the likelihood of detecting study organisms. Given the small flock sizes of our study farms (10–500 birds), sampling five birds per farm was considered representative of the flock.

For the isolation of *E*. *coli*, cloacal swabs were placed in Amies without charcoal, while for the *Campylobacter* spp. swabs were placed in Amies transport medium with charcoal. All samples were appropriately labelled and transported under cold chain at 4 ℃ to the bacteriology laboratory, at the Department of Veterinary Pathology, Microbiology and Parasitology, University of Nairobi. Samples were delivered to the laboratory within 2–4 h of collection for further microbiological analysis.

### Bacterial isolation and identification

For bacterial isolation, the chicken cloacal swab samples were cultured using conventional culture and isolation according to Markey et al. [30]. For *E*. *coli* isolation, all five cloacal swabs from the same farm were pooled and homogenized in 9 mL of freshly prepared buffer peptone water (BPW). The resulting homogenate was used as one composite flock-level sample for *E*. *coli*. The resultant homogenate was then streaked aseptically onto MacConkey agar (Oxoid, Basingstoke, UK) and incubated aerobically at 37 ℃ for 18 h. Presumptive identification of the *E*. *coli* was done using colony morphology, Gram staining reaction, and a comprehensive biochemical analysis.

Similarly, for isolation of *Campylobacter* spp., all five cloacal swabs from the same farm were pooled and homogenized in 9 mL of freshly prepared buffer peptone water (BPW). The resulting homogenate was used as one composite flock-level sample for *Campylobacter*. The suspension was transferred to Preston broth for selective enrichment and incubated at 42 °C for 24 h. After which the broths were streaked aseptically onto modified charcoal-cefoperazone-deoxycholate agar (mCCDA) (Oxoid, Basingstoke, UK) plates (incorporated with *Campylobacter* selective supplement), which were further incubated microaerophilic condition (5% O_2_, 10% CO_2_, 85% N_2_) using anaerobic jars equipped with microaerobic gas-generating bags (Thermo Scientific™, Waltham, MA, USA) at 42 °C for 48 h. Distinct colonies were sub-cultured to obtain pure colonies by re-streaking on blood agar plates (with selective supplement). Presumptive identification of the *Campylobacter* suspect colonies was done by culture characteristics (growth at 42 °C) and colony morphology.

Furthermore, species-level confirmation of *E*. *coli* and *Campylobacter* spp. was performed using matrix-assisted laser desorption/ionization time-of-flight mass spectrometry (MALDI-TOF MS) (Bruker Daltonics, Germany), applying standard Bruker interpretative criteria at the Central Veterinary Laboratory, Kabete. Briefly, scores of ≥ 2.0 were considered reliable for species-level identification, scores of ≥ 1.7 to < 2.0 for genus-level identification, and scores < 1.7 were deemed unreliable.

### Antimicrobial Susceptibility Testing (AST)

Antimicrobial susceptibility testing for 104 *E*. *coli* and 104 *Campylobacter* spp. isolates were performed using the Kirby-Bauer disk diffusion method, as per Clinical and Laboratory Standards Institute guidelines [31, 32]. The isolates were selected using a representative sampling approach by sub-county and chicken production type (broilers, layers, and indigenous chickens) to ensure proportional representation across the sampled poultry populations. Briefly, for *E*. *coli*, fresh colonies were emulsified into sterile saline to achieve turbidity equivalent to 0.5 McFarland standard. Suspensions were spread onto Muller Hinton agar (MHA) (Oxoid, Basingstoke, UK) using sterile cotton swabs and incubated aerobically at 37 °C for 18 h as per the guidelines. Ten antimicrobials of veterinary and medical importance were used to determine the susceptibility of the isolates. These included; ampicillin (AMP) (10 µg), amoxicillin-clavulanic acid (AMC) (20/10µg), tetracycline (TET) (30 µg, ) imipenem (IMP) (10 µg), gentamicin (GEN) (10 µg), cefotaxime (CTX) (30 µg), ciprofloxacin (CIP) (5 µg), streptomycin (STR) (10 µg), ceftazidime (CAZ) (30 µg), trimethoprim/sulfamethoxazole (1.25/23.75 µg) (STX) (Oxoid Ltd., Basingstoke, UK). The choice of antimicrobial tested was guided by priority antimicrobials monitored under the Kenya national antimicrobial resistance surveillance strategy 2023–2027 and the WHO bacterial priority list [33]. *Escherichia coli* ATCC 25,922 was used as the quality control strain as per the CLSI guidelines.

For screening and confirmation of Extended-spectrum beta-lactamase (ESBL) production in *E*. *coli*, a combined disk test was done as per the CLSI guidelines [31]. Briefly, the suspected ESBL isolates were confirmed using ceftazidime (CAZ 30 µg), cefotaxime (CTX 30 µg), ceftazidime/clavulanic acid (CAZ/CLA 30/10 µg), and a disk with and without cefotaxime/clavulanic acid (CTX/CLA 30/10 µg). After incubation at 37 °C for 18 h, the diameter was measured and recorded. The isolates were ESBL-positive when an increase of ≥ 5 mm in the zone diameter of any of the antibiotic disks in combination with clavulanic acid. While an increase < 5 mm in the zone diameter, the result was considered ESBL-negative. For quality control, *E*. *coli* ATCC 25,922 and *Klebsiella pneumoniae* ATCC 700,603 were used.

Briefly, a confirmed pure and fresh *Campylobacter* colony was suspended on 0.2% sterile normal saline and standardized to a 0.5 McFarland turbidity index using a Nephelometer. The resulting suspension was then streaked aseptically using a sterile swab onto dried Muller Hinton agar (Oxoid Ltd., Basingstoke, UK) supplemented with 5% defibrinated sheep blood (MHBA) plates within 15 min of standardization. Four antimicrobials, namely CIP = ciprofloxacin (5 µg), ERY = erythromycin (15 µg), NAL = nalidixic acid (30 µg), and TET = tetracycline (30 µg) (Oxoid Ltd., Basingstoke, UK) were used. The choice of antimicrobial tested was guided by priority antimicrobials monitored under the Kenya national antimicrobial resistance surveillance strategy 2023–2027. Further, the selection of the four antimicrobials tested against *Campylobacter* spp. was guided by the CLSI M45 guidelines [32], considering the availability of antimicrobial disks and the presence of established interpretive breakpoints. The plates were then incubated in the microaerophilic conditions (5% O_2_, 10% CO_2_, 85% N_2_) using anaerobic jars equipped with microaerobic gas-generating packs (Thermo Scientific™, Waltham, MA, USA) for 24 h at 42 °C. *Staphylococcus aureus* ATCC 25,923 was used as the quality control strain as per the CLSI guidelines [32].

The diameters of inhibition zones for both isolates were measured using a Vernier calliper, and the results were recorded. Interpretation of the results for *E*. *coli* and *Campylobacter* spp. was then done according to the Clinical Laboratory Standards Institute [31, 32]. Based on the zones of inhibition, isolates were classified as either resistant (R), intermediate (I), or susceptible (S) to each antibiotic tested. In this study, all intermediates were grouped as the Resistant (R) category. Multidrug resistance (MDR) was defined as resistance to at least one antimicrobial agent in three or more antimicrobial classes.

### Data analysis

The data entry and management were done using Microsoft Excel 2024. Data analysis was done using R Studio (version 4.4.0). Descriptive statistics were used to calculate the proportions and frequencies of all variables. Prevalence was obtained as the proportion of total positive samples over the total samples. To check for association between sub-county, chicken type, and the number of antimicrobials to which isolates were resistant, the non-parametric Kruskal–Wallis test was applied. Pearson correlation analysis was also conducted to examine the relationship between antimicrobial usage and resistance profiles across sub-counties. MDR was defined as resistance to at least three antimicrobials from three different classes. Statistical significance for this study was set at 0.05 (*p* < 0.05).

## Results

### The distribution of the chicken sampled

**Fig. 2:**
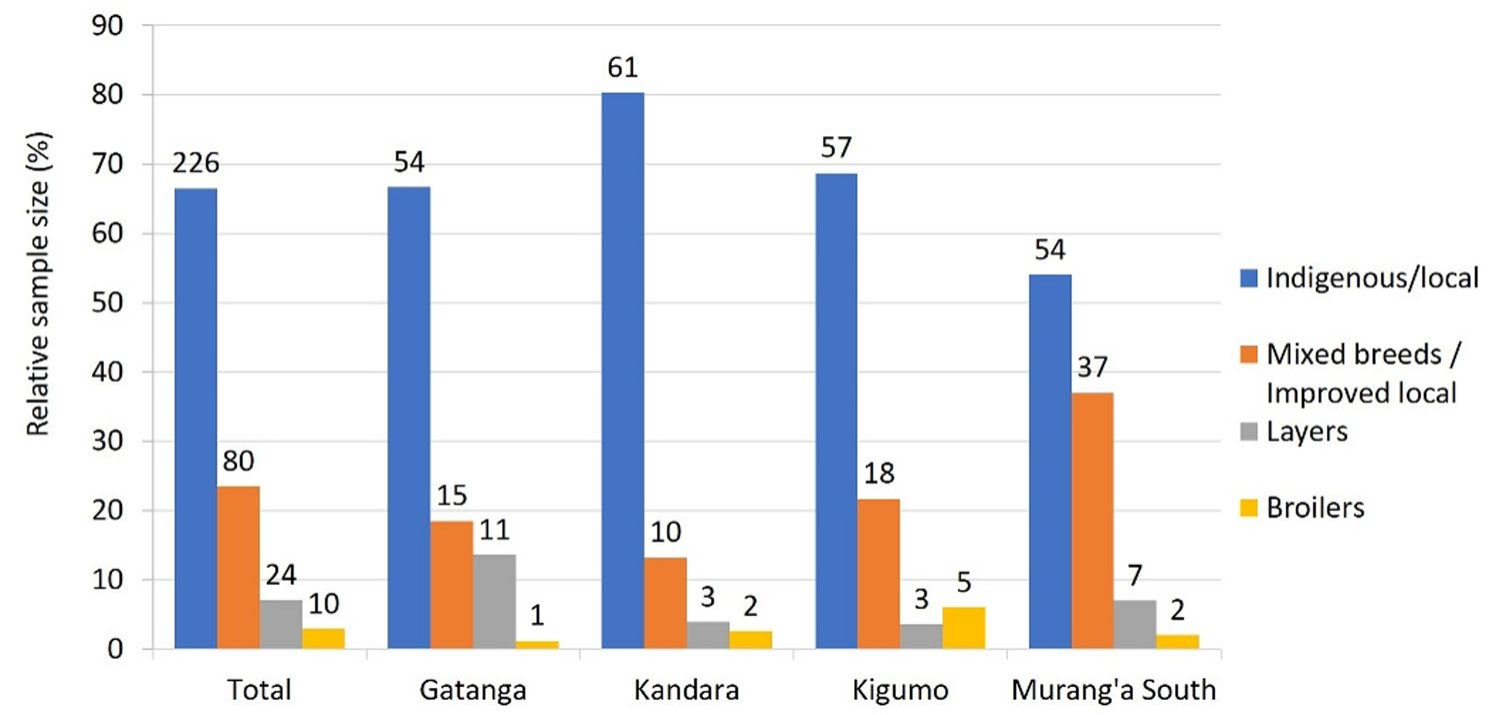
Distribution of chickens sampled based on sub-county and chicken type in Murang’a County, Kenya. The number of samples (n) is indicated on top of each bar

A total of 340 pooled cloacal swab samples collected from chickens reared in small-scale poultry farms in four sub-counties of Murang’a County, Gatanga, Kandara, Kigumo, and Murang’a South, were analysed in this study. The distribution of the chicken sampled and chicken types from each sub-county is shown in Fig. 2. Overall, across all sites, local/indigenous chickens were the predominant type sampled at 66.5% (*n* = 226/340), improved/mixed-breed chickens at 23.5% (*n* = 80/340), with the least being broilers at 2.9% (*n* = 10/340). Sub-county-level data analysis of chicken samples showed variation in the representation of chicken types and number; however, the sampling was done based on the number of chickens and representation of each type at each sub-county (Fig. 2).

### Antimicrobial usage in small-scale poultry farms in Murang’a County

**Fig. 3:**
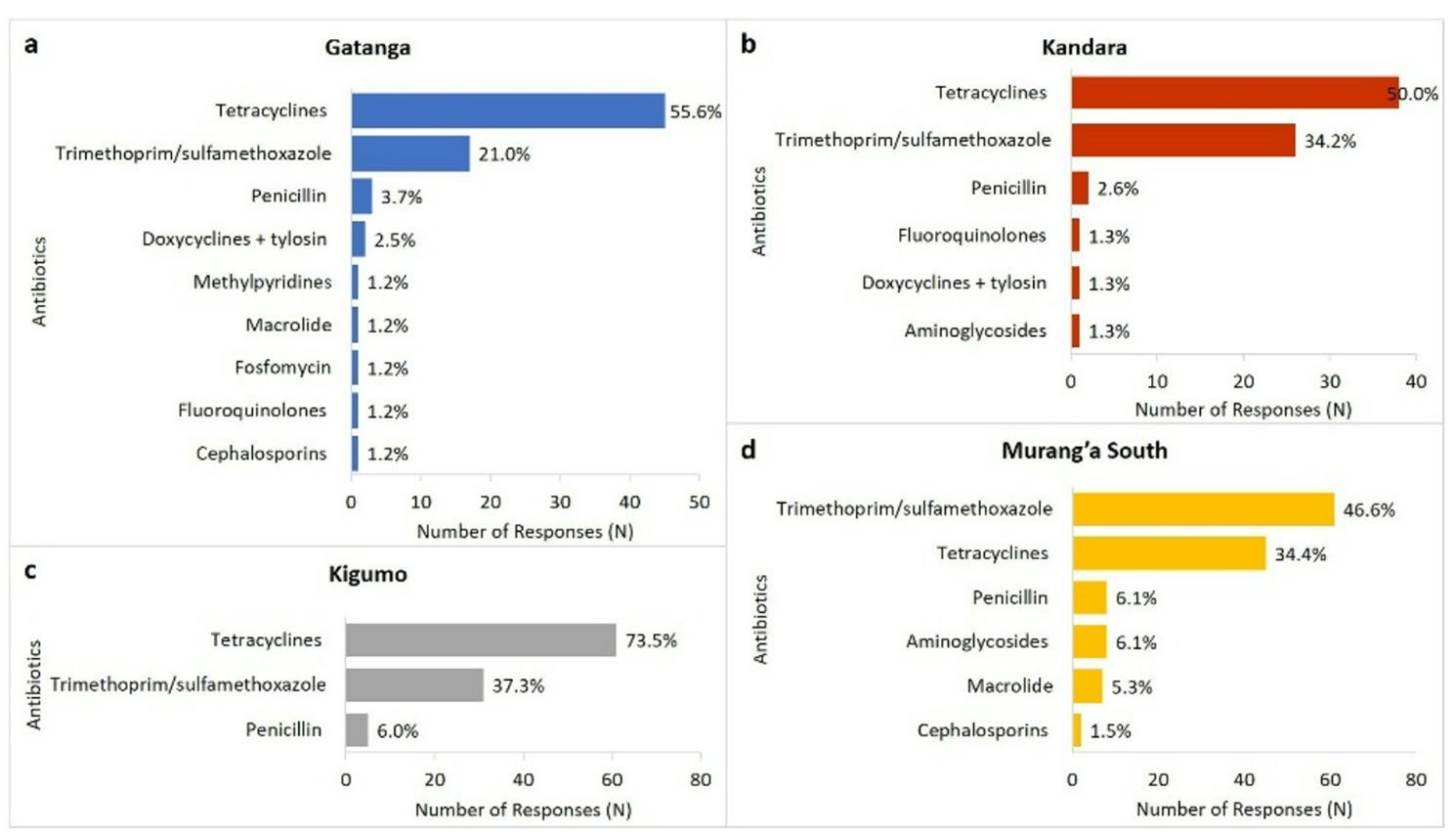
Distribution of commonly used antimicrobial classes in a small-scale chicken production system in four sub-counties (**a**, **b**, **c**, **d**) of Murang’a County, Kenya

Of the 340 poultry farms, 75.3% (*n* = 256/340) across all sub-counties reported using antimicrobials in poultry production. Among the farms that reported antimicrobial use, the primary purposes included treatment of sick birds 94.5% (*n* = 242/256), disease prevention 54.3% (*n* = 139/256), and growth promotion 4.7% (*n* = 12/256). Multiple responses were allowed, as some farms reported using antimicrobials for more than one purpose. Usage was highest in farms in Kigumo at 85.5% (*n* = 71/83), followed by in Murang’a South 84.0% (*n* = 84/100), and least was in Kandara at 60.5% (*n* = 46/76) farms. As shown in Fig. 3, tetracyclines were the most commonly used antimicrobials (73.8%, *n* = 189) in poultry production in Murang’a County. Across the sub-counties, farms in Kigumo had the highest reported use of antimicrobials (73.5%), followed by Gatanga (55.2%), and Murang’a South (45.0%), as shown in Fig. 3. Trimethoprim/sulfamethoxazole was the second most frequently used antimicrobial overall at 52.3% (*n* = 134/256), with the highest usage reported in Murang’a South (61.0%), and lower usage in Gatanga (20.5%). Use of some critically important and newer generation of antimicrobials, such as aminoglycosides, macrolides, cephalosporins, fosfomycin, and fluoroquinolones, was reported at relatively lower prevalence, ranging at 0.3%- 8% (Fig. 3).

### Occurrence of *E*. *coli* and *Campylobacter* spp. from the chicken cloacal swabs

Isolation of *E*. *coli* and *Campylobacter* species from the 340 pooled cloacal swabs were 99.4% (*n* = 338/340) for *E*. *coli*, 36% (*n* = 123/340) for *Campylobacter jejuni*, and 25.3% (*n* = 86/340) for *Campylobacter coli*. The differences in *E*. *coli* occurrence across sub-counties were not statistically significant (χ² = 2.35, *p* = 0.503). However, there were notable differences in the isolation rate of *Campylobacter* species. *Campylobacter coli* was highest in cloacal swabs from Gatanga at 37% (*n* = 30/81), compared to 22.4% (17/76) in Kandara, 20.5% (*n* = 17/83) in Kigumo, and 22% (*n* = 22/100) in Murang’a South, with the variation being statistically significant (χ² = 64.27, *p* < 0.001). Similarly, *C*. *jejuni* prevalence varied considerably, with cloacal swabs from Kigumo reporting the highest rate at 56.6% (*n* = 47/83), followed by Gatanga at 38.3% (*n* = 31/81), Murang’a South at 28% (*n* = 28/100), and Kandara at 22.4%(*n* = 17/38). Co-isolation of *C*. *jejuni* and *C*. *coli* was rare, with only 6 samples having both species. 141 samples did not have *Campylobacter* growth.

### Prevalence of antimicrobial-resistant *E*. *coli*

**Fig. 4:**
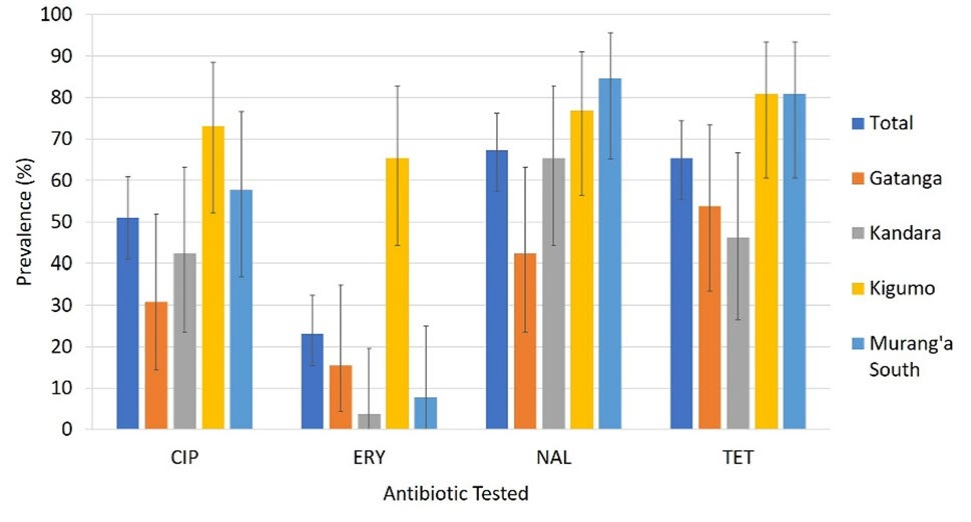
Prevalence of antimicrobial-resistant *Campylobacter* species based on region (study sub-county). The error bars indicate the 95% CI. CIP = ciprofloxacin, ERY = erythromycin, NAL = nalidixic acid, and TET = tetracycline

A total of 104 *E*. *coli* isolates were selected and tested for antimicrobial resistance using the Kirby-Bauer disc diffusion method. Isolates were selected through stratified sampling based on county of origin and chicken type to ensure proportional representation of the poultry populations. Overall, 93.2% (*n* = 97/104) *E*. *coli* were resistant to at least one of the antimicrobials tested. Among the *E*. *coli*, as shown in Table 1, the most common resistance was observed against tetracycline at (74%), followed by trimethoprim/sulfamethoxazole (65.4%), ampicillin (51.9%) and the least was cefotaxime at 3.8%. Notably, significant phenotypic resistance to ciprofloxacin (19%), imipenem (25%), and ceftazidime (10.6%) was reported in this study (Fig. 4). Only 4.8% (*n* = 5/104) isolates were ESBL-positive, all from the local /indigenous breeds. Based on sub-county, isolates from Murang’a South showed the highest resistance to ampicillin (73.1%), gentamicin (73.1%), and ceftazidime (26.9%), while notably isolates from Gatanga showed no resistance to gentamicin, cefotaxime, or ceftazidime. *E. coli* isolates from Kandara recorded the highest resistance to tetracycline (84.6%) and sulphamethoxazole-trimethoprim (80.8%), and no resistance to cefotaxime Table 1.

**Table 1:** Prevalence of antimicrobial-resistant *Escherichia coli* isolated from poultry in small-scale farms in Murang’a County, Kenya (*n* = 104)

Antibiotic	Total (%) (*n* = 104)	Sub-County			
		Gatanga (%) (*n* = 26)	Kandara (%) (*n* = 26)	Kigumo (%) (*n* = 26)	Murang'a South (%) (*n* = 26)
AMC	7.7	0.0	3.8	7.7	19.2
AMP	51.9	38.5	53.8	42.3	73.1
CAZ	10.6	0.0	7.7	7.7	26.9
CIP	19.2	3.8	11.5	23.1	38.5
CTX	3.8	0 .0	0.0	3.8	11.5
GEN	25.0	0 .0	7.7	19.2	73.1
IMP	26.0	53.8	42.3	0.0	7.7
STR	39.4	30.8	42.3	34.6	50
SXT	65.4	46.2	80.8	69.2	65.4
TET	74.4	61.5	84.6	80.8	69.2

*AMC* Amoxicillin-clavulanic acid, *AMP*  Ampicillin, *CAZ*  Ceftazidime, *CIP*  Ciprofloxacin, *CTX*  Cefotaxime, *GEN*  Gentamicin, *IMP*  Imipenem, *STR* Streptomycin, *SXT*  Trimethoprim/sulfamethoxazole and *TET*  Tetracycline

Based on chicken types, *E. coli* isolates from local chickens (*n* = 69) exhibited the highest resistance to tetracycline (49%), trimethoprim/sulfamethoxazole (44%), and the least was cefotaxime at 3%. Notably, resistance to amoxicillin-clavulanic acid and cefotaxime was not observed in isolates from layers and broilers (Table 2).

**Table 2:** Prevalence of antimicrobial-resistant *E*. coli based on chicken type in small-scale poultry farms of Murang’a County, Kenya

Antibiotic	Chicken type			
	Indigenous/local (%) (*n*=69)	Mixed breeds / Improved local (%) (*n*=26)	Layers (%) (*n*=8)	Broilers (%) (*n*=1)
AMC	6	2	0	0
AMP	35	12	7	0
CAZ	9	2	0	0
CIP	11	7	2	0
CTX	3	1	0	0
GEN	15	11	0	0
IMP	18	5	4	0
STR	30	8	3	0
SXT	44	17	6	1
TET	49	20	7	1

*AMC* Amoxicillin-clavulanic acid, *AMP* Ampicillin, *CAZ* Ceftazidime, *CIP* Ciprofloxacin, *CTX* Cefotaxime, *GEN* Gentamicin, *IMP* Imipenem, *STR* Streptomycin, *SXT* Trimethoprim/sulfamethoxazole and *TET* Tetracycline

The overall *Campylobacter* spp. isolates showed the highest resistance to nalidixic acid (67.3%), followed by tetracycline (65.4%), and least to erythromycin (23.1%) (Fig. 4). Across sub-counties, isolates from Kigumo recorded the highest levels of resistance across the four antimicrobials tested at nalidixic acid (76.9%), tetracycline (80.8%), and erythromycin (65.4%), as shown in Fig. 4. This was followed by *Campylobacter* isolates from Murang’a South, and the least resistance across the 4 antimicrobials was reported in isolates from Gatanga, as per Fig. 4.

Based on chicken type, *Campylobacter* spp. isolates showed high resistance to nalidixic acid across all groups, with the highest resistance observed in layer chicken (81.8%) and the lowest (50.0%) in broilers (Table 3). Notably, the *Campylobacter* isolates from layer chickens showed high resistance to tetracycline (72.7%) and ciprofloxacin (63.6%), followed by mixed breeds (65% and, 63% respectively) (Table 3). Overall, lower resistance to erythromycin was observed among isolates from different chicken types, with a prevalence of 50.0% in broilers and 27.3% in layers (Table 3).

**Table 3:** Prevalence of antimicrobial-resistant *Campylobacter* species based on chicken type in small-scale farms of Murang’a County, Kenya

Antibiotic	Chicken type			
	(%) (*n* = 65)	Mixed breeds / Improved (%) (*n* = 26)	Layers (%) (*n* = 11)	Broilers (%) (*n* = 2)
CIP	50.0	50.0	63.6	0.0
ERY	23.1	19.2	27.3	50.0
NAL	67.7	61.5	81.8	50.0
TET	63.1	65.4	72.7	1.00

*CIP*  Ciprofloxacin, *ERY*  Erythromycin, *NAL*  Nalidixic acid, and *TET*  Tetracycline

**Fig. 5:**
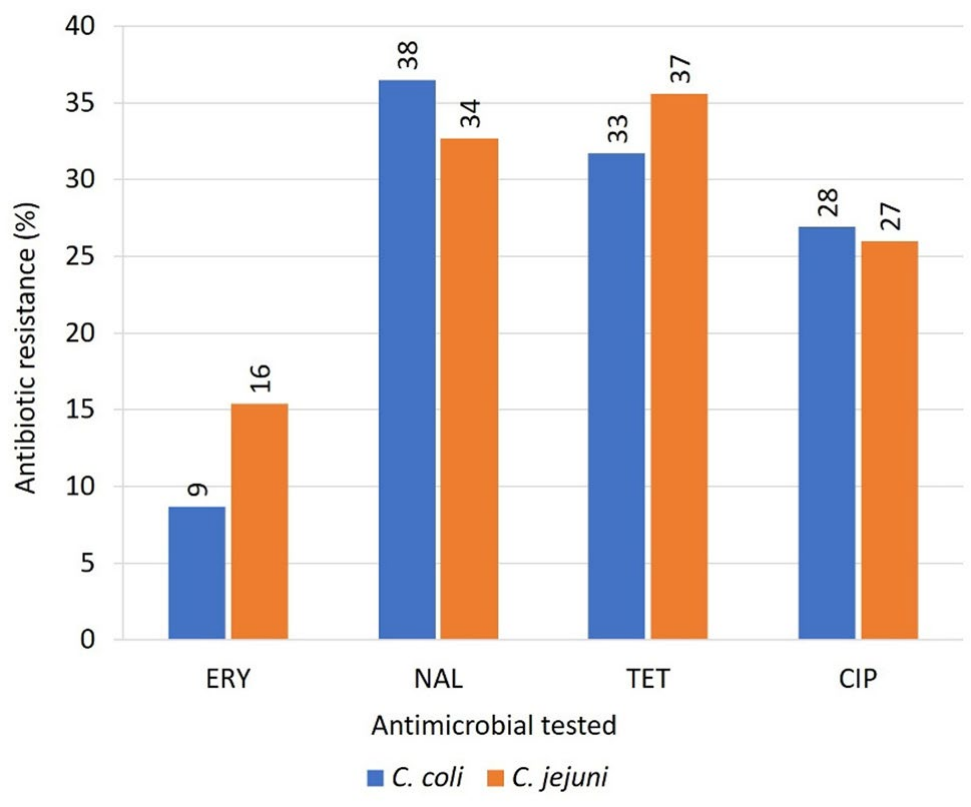
Distribution of antimicrobial resistance among *Campylobacter* species (*n* = 104) isolates from Murang’a County, Kenya. Antibiotics tested included ciprofloxacin (CIP), erythromycin (ERY), nalidixic acid (NAL), and tetracycline (TET)

As shown in Fig. 5, among the *Campylobacter* species, overall higher resistance to tetracycline, ciprofloxacin, and erythromycin was reported in *C*. *jejuni* compared to *C*. *coli*. However, resistance to nalidixic acid was higher in *C*. *coli* (34.6%) compared to *C*. *jejuni* (30.8%).

### Multidrug resistance among isolates from chicken from small-scale poultry farms in Murang’a County, Kenya

**Fig. 6:**
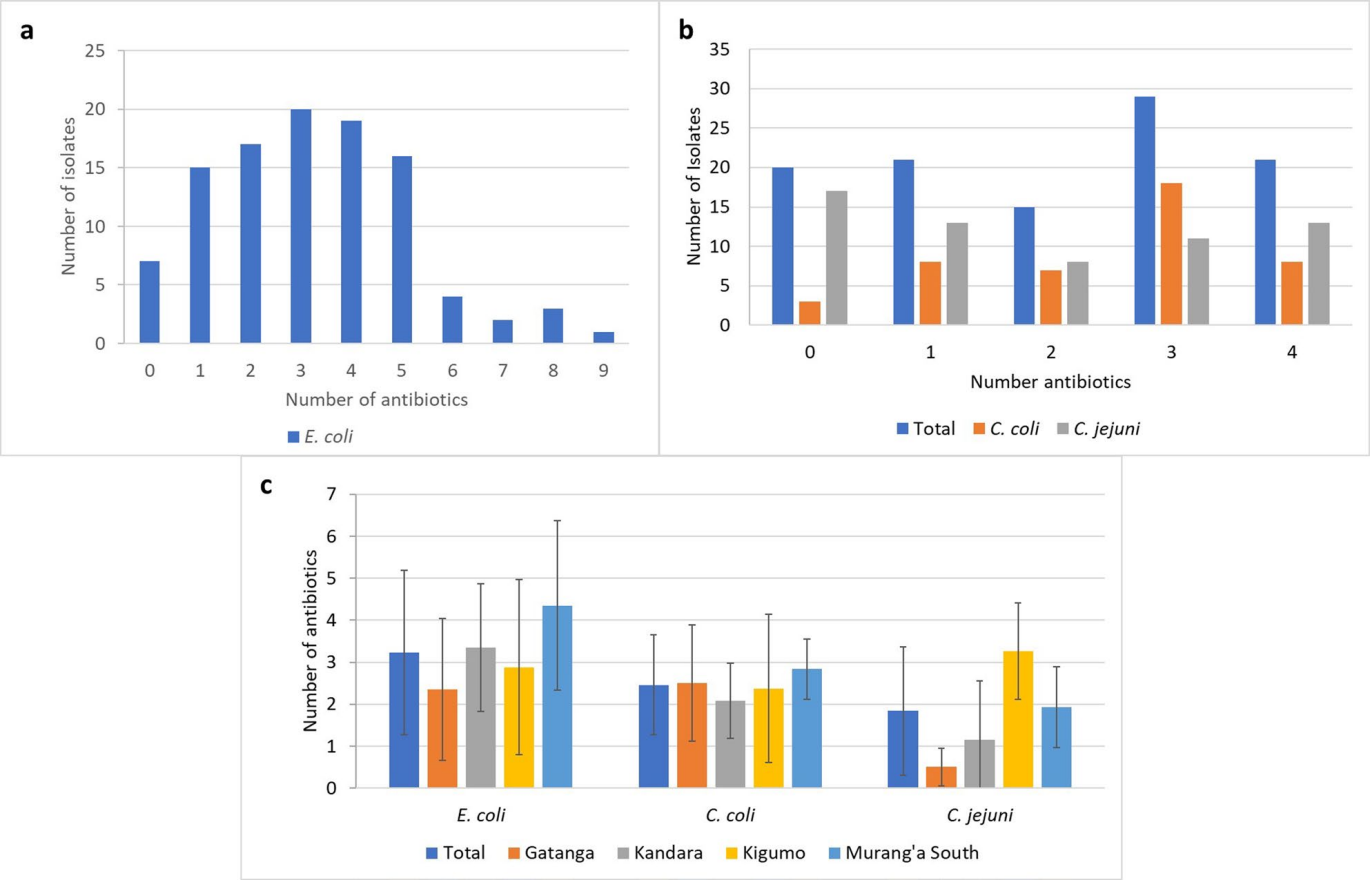
Average number of antimicrobial resistance observed. **a**, **b** Number of isolates resistant to different antimicrobials per organism. **c** Mean antimicrobial resistance by organism and sub-county

Multidrug-resistance (MDR) was observed in 58.6% (*n* = 61/104) of *E*. *coli* and 46.1% (*n* = 48/104) of *Campylobacter* spp. isolates. Fig. 6 (a, b) presents the average number of antimicrobials to which the isolates were resistant. Among *E. coli* isolates, the most common resistance combinations involved penicillins, aminoglycosides, cephalosporins, and fluoroquinolones, with four isolates exhibiting resistance to at least eight of the ten antimicrobials tested.

For *Campylobacter* spp., the highest number of resistant isolates showed resistance to three antimicrobials (*n* = 28), most commonly nalidixic acid, tetracycline, and ciprofloxacin. Notably, (*n* = 20) isolates were resistant to all four antimicrobials tested.

#### Association of AMR resistance level with subcounty and chicken types

The non-parametric Kruskal-Wallis test conducted to determine whether sub-county and chicken type were associated with the number of antimicrobials to which *E*. *coli* isolates were resistant indicated that neither the chicken type (H = 1.08, *p* = 0.782) nor the sub-county significantly influenced resistance levels. Similarly, no significant difference in resistance counts was observed across different chicken types (H = 1.38, *p* = 0.709 and sub-counties (H = 6.81, *p* = 0.078) for *Campylobacter*.

### Correlation between antimicrobial use and antimicrobial resistance

Assessment of the correlation between antimicrobial use and resistance profiles for *E*. *coli* and the *Campylobacter* spp. was assessed through Pearson correlation (Table 4). The Pearson correlation coefficient in Kigumo, for *E*. *coli*, was 0.72 with a significant p-value of 0.04. Gatang’a and Kandara showed correlations of 0.57 (*p* = 0.14) and 0.80 (*p* = 0.10), respectively, while Murang’a South had the lowest correlation at 0.21 (*p* = 0.62). For *Campylobacter*, no statistically significant correlation was observed. However, the highest correlation was observed in Gatang’a (*r* = 0.45, *p* = 0.26), and the lowest in Murang’a South (*r* = 0.03, *p* = 0.95).

**Table 4:** Pearson Correlation coefficients (r) for antimicrobial usage and antimicrobial resistance profiles among the two test organisms for various antibiotic classes in the sampled sub-counties, Murang’a County, Kenya

Sub county	*Escherichia coli*		*Campylobacter* spp.	
	*R*	*p* Value	*R*	*p* Value
Gatanga	0.57	0.14	0.45	0.26
Kandara	0.80	0.10	0.07	0.87
Kigumo	0.72	**0.04**	0.15	0.72
Murang’a South	0.21	0.62	0.03	0.95

## Discussion

The present study reported a high (75%) frequency of antimicrobial use in poultry farming in the study area, particularly tetracyclines and sulfonamides. These findings align with previous studies on widespread use of antimicrobials in small-scale poultry chicken farming in different regions in Kenya [14, 20, 22, 34]. However, significantly higher prevalence has been reported in Uganda at 95% [35], Zambia at (83%) [36], and Ghana (97.0%) [37] in domestic and commercial poultry farms. Although our findings are lower than what has been reported from neighbouring countries, the prevalence reported remains substantial and could be attributed to increasing demand for poultry products, weak antimicrobial use regulation in food animals, inadequate farm biosecurity, and limited AMR awareness among farmers [38]. These findings highlight the critical need for contextualized interventions such as enhanced antimicrobial stewardship programs and improved farm biosecurity measures to reduce antimicrobial use in poultry farms in Kenya.

Tetracyclines and sulphonamides were the most commonly used antimicrobial classes in the study areas. A recent study in Kenya focusing on semi-intensive broiler farms reported tetracycline and erythromycin as the most commonly used antimicrobials [39]. Use of these antimicrobials either singly or in combinations for therapeutic and prophylactic purposes without veterinary guidance has been reported in Kenya previously [14, 20, 22, 34].

In addition, our study found that, although at lower prevalence, other critically important, last resort antimicrobials, such as fluoroquinolones, cephalosporins, and fosfomycin (0.34-8%), were also used. These findings align with previous studies in Kenya [12, 17, 24], Ghana [37], and Pakistan [40]. Most of these drugs are highly restricted and not licensed for use in food animals [39], suggesting unauthorized channels of entry or source into the Kenyan Markets. These findings support the urgent need for farmers to be educated on AMR and proper biosecurity measures to avoid misuse of antimicrobials. Furthermore, there is a need to strengthen and enforce national guidelines on antimicrobial use and access in food animals in Kenya [39].

The recovery rate of *E*. *coli* in this study was 99.4%, which is consistent with its role as a ubiquitous commensal organism in the intestinal microbiota of poultry. This agrees with other studies in Kenya [41], Australia [42], and Zambia [43], where isolation of 90%- 100% *E*. *coli* has been reported in chicken cloacal swabs. However, it differs from other studies in Kenya on indigenous chicken that reported lower prevalence of 85.5% [44], 67% [24], 57% [25], and 31.4% [26]. *E. coli* is a commensal organism mostly found in normal flora in the intestines and faeces of animals and humans [45, 46]. Differences in recovery rate of *E*. *coli* could be due to variation in laboratory methodologies and epidemiological designs, such as chicken type, production types, geographical regions, and farm biosecurity practices. Further in our study, flock-level pooling might have increased the likelihood of *E. coli* detection [47].

*Campylobacter* species were isolated at a prevalence of 61.4%, with *C*. *jejuni* at 36.1% and *C*. *coli* at 25.3%. The prevalence of *Campylobacter* spp. in cloacal swabs has been reported to vary widely across studies, ranging from 7.71% to 77.6%, depending on study location, sample type, and the specific species. The prevalence reported in the current study is within the range of other similar studies in Kenya [48], Tanzania [49], Ethiopia [50], Spain [51], and Malaysia [52]. However, higher prevalence of *Campylobacter* has been reported at 93% in commercial farms in the USA [53], 85.2% in broiler chicken in North west Romania [54], and 99% in the poultry food chain in Canada [9]. Our study reported a higher dominance of *C*. *jejuni* over *C*. *coli*; however, a lower frequency of isolation of *C*. *coli* as the most predominant species in chicken has been reported in Ghana [37] and Ethiopia [55]. Further, higher dominance of *C*. *coli* towards *C*. *jejuni* (63.4% vs. 36.6%) has also been reported in Romania [54]. An adjusted prevalence of approximately 40% for *Campylobacter* spp. in poultry has been reported in a meta-analysis covering 27 African countries [13]. This reflects the endemic nature of *Campylobacter* spp. in the chickens and is potentially increased by other external farm-level contamination, enhancing flock colonization [13, 54, 56]. Stringent biosecurity measures have been shown to reduce the prevalence of *Campylobacter* spp. in chicken farms, and therefore should be enhanced in Kenyan production systems [56].

In this study, 92.3% of *E*. *coli* were resistant to at least one of the antimicrobials tested, consistent with findings from other studies in Kenya [25, 26]. Resistance was highest to tetracycline (74%), sulphamethoxazole–trimethoprim (65.4%), and ampicillin (51.9%). Other studies in Kenya [26] reported similar resistant patterns in ampicillin (85.22%), tetracycline (66.7%), and co-trimoxazole (57.4%). Similar AMR patterns were also reported in Uganda [57], where higher resistance to ampicillin 79.8%, tetracycline 72.8% and cotrimoxazole 55.7% were observed. However, relatively lower resistance in *E*. *coli* to the same antimicrobials, tetracycline (41%), trimethoprim/sulfamethoxazole (32%), and ampicillin (17%) has been reported among isolates from various livestock, including chicken in Kenya [58]. This finding likely reflects the widespread use and misuse of these antimicrobials in poultry farming. Consistent with this, our antimicrobial use data indicate that tetracyclines and sulphonamides were the most commonly used classes, with oxytetracycline and sulphonamides among the most sold drugs to poultry farmers in Kenya [17, 18]. Use of combination antimicrobials (more than one molecule) in poultry production has also been reported [14, 20]. Antimicrobial use in poultry farming in Kenya must be regulated to reduce AMR in bacteria from poultry farms and food animals.

The detection of phenotypic resistance to critically important antimicrobials such as fluoroquinolones, carbapenems, and third-generation cephalosporins reported in this study raises significant public health concerns. Similar findings have been reported in Uganda by Nyolimati et al. [57], where cefepime and ceftriaxone were both at (7%) and imipenem (11.6%), with higher levels of resistance to ciprofloxacin at 38%. Most of these drugs are highly prohibited for use in food animals in most countries, including Kenya, as they are under the watch and reserve category for human health [33]. Therefore, further studies should be done to find out the sources of this resistance.

Notably, the high level of phenotypic resistance to carbapenems observed in this study is of significant public health concern and warrants further investigation. Lower prevalence (15.9%) has been reported in chickens in Nairobi, Kenya [59], while another recent study in Kenya reported no detectable carbapenem resistance in poultry [23]. Given that carbapenems are not licensed for use in food animals in Kenya, the observed resistance may reflect alternative drivers, including environmental or human-associated sources. Indeed, a high prevalence (68%) of carbapenem-resistant *Enterobacteriaceae* has been reported from Nairobi River surface water [60], suggesting potential environmental reservoirs. Although this may reflect localized contamination, cross-transmission between the environment, animals, and humans cannot be excluded. Therefore, further studies adopting a One Health approach are needed to investigate the sources, transmission pathways, and drivers of carbapenem resistance in the study area.

In this study, *Campylobacter* spp. isolates showed the highest resistance to nalidixic acid at 67.3%, followed by tetracycline at 65.4%, and ciprofloxacin at 51.8%, with minimal variation within the study sites and species. These findings agree with other previous studies in *Campylobacter* in Kenya, where *C*. *jejuni* isolates showed a high rate of resistance to nalidixic acid, tetracycline, and ciprofloxacin of 77.4%, 71.0%, and 71.0%, in backyard chicken in Thika [61]. Another study in Kenya [22] found a higher resistance to tetracycline (97.1%) and ciprofloxacin (63.1%). A recent study in East Africa also reported a similar trend of *Campylobacter* species from chicken with nalidixic acid, tetracycline, and ciprofloxacin at 63.9%, 66% 63.9% respectively [62]. However, slightly lower resistance to ciprofloxacin (40.2%), tetracycline (46.6%), and nalidixic acid (49.2%) has been reported in the USA [63, 64]. With even lower resistance to fluoroquinolones and tetracyclines being reported in the USA in farms where chickens are raised without antimicrobials [64]. Widespread use of fluoroquinolones and tetracyclines in poultry production has been reported in Kenya and other African countries and may contribute to the high level of resistance observed [48–50].

This study reported a lower prevalence of resistance to erythromycin (24.5%), similar to findings reported in Ethiopia (16.3%) [55]. Higher resistance to erythromycin, 75.7% among *Campylobacter* isolates, has previously been reported in Kenya [22]. However, significantly lower prevalence at 5.5% has been reported in East African poultry studies [62]. The variation in resistance level could be attributable to different production systems and poultry breed types [55]. The present study focused on indigenous (local) and mixed (crossbred) chicken breeds. The findings indicate a relatively lower prevalence of resistance to macrolides; however, continued monitoring is necessary to prevent overuse.

In this study, 58.6% of the *E*. *coli* and 46.1% of *Campylobacter* species were multidrug resistant (MDR). Higher MDR levels have been previously reported in Kenya (81.5%) in *E*. *coli* [44], *Campylobacter* (61.3%) [61], and *Campylobacter* spp. (96.1%) [22] previously. Similarly, higher prevalence of MDR *E*. *coli* has also been reported in other countries such as Tanzania [65] and Uganda [57]. However, significantly lower prevalence of MDR *Campylobacter* (9.3%) has been reported in Ethiopia [55]and 4.7% in Ghana [37]. The majority of MDR combinations in *E*. *coli* were in 5 classes of critical medicines for human health. The high MDR reported could be due to widespread antimicrobial use in chicken production that has been reported in Kenya previously [14, 20, 34]. This finding highlights the increasing MDR from food animals, posing a significant public health concern as the MDR bacteria could end up in the food chain and the environment [65].

Unexpectedly, in this study, despite widespread antimicrobial use—including critically important antimicrobials for human medicine—no direct statistical association was observed between reported antimicrobial use and resistance profiles. Except in *E*. *coli*, resistance profiles in Kigumo sub-county. This finding may reflect limitations such as self-reported antimicrobial use data, unmeasured historical antimicrobial exposure, environmental reservoirs, unquantified exposure, or complex resistance drivers beyond direct use. Future studies should include longitudinal data, detailed antimicrobial usage metrics, and molecular analyses to clarify the relationship between antimicrobial use and resistance in these regions.

### Study limitation

This study had some limitations that should be considered when interpreting the findings. First, antimicrobial use data were obtained through farmer self-reports using questionnaires, which may be subject to recall bias. Secondly, the cross-sectional design captures practices and resistance patterns at a single point in time and therefore does not allow causal relationships to be established. Thirdly, cloacal swabs from multiple birds were pooled at the farm level to represent flock-level bacterial carriage and improve detection; however, this approach may reduce the ability to capture within-flock variability. The small sample size for some chicken types, particularly broilers and layers, and the focus on a single study county may limit broader generalization of these results. Further, the detection of AMR was only done phenotypically, and therefore, future studies should consider whole-genome characterization to determine the resistance mechanism. Despite these limitations, this study provides valuable baseline data for Central Kenya and contributes to the growing body of evidence on AMR in low- and middle-income countries.

## Conclusion

This study found a high prevalence of antimicrobial-resistant *E*. *coli*, *C*. *jejuni*, and *C*. *coli* in small-scale poultry systems. The co-occurrence of *E*. *coli* and *Campylobacter* spp. in chickens heightens the zoonotic risks associated with poultry production systems. Additionally, high antimicrobial usage, including some critically important antimicrobials for human health, although not directly correlating to AMR in this study, underscores the need for continued monitoring and regulation. Our study findings highlight the urgent need for context-specific interventions to reduce the high prevalence of AMR and MDR *E*. *coli* and *Campylobacter* spp. in indigenous/mixed-breed chicken production in Kenya, particularly in rural settings. Strengthening antimicrobial stewardship and developing targeted strategies to reduce AMR bacteria in food animals in Kenya is urgent. Future studies should adopt a One Health approach, incorporate human and environmental samples, and investigate risk factors associated with antimicrobial use and MDR in *E*. *coli* and *Campylobacter* spp.

## Supplementary Information

Supplementary Material 1. Supplementary File S1: Structured questionnaire used in the study - Assessment of Antimicrobial Use and Resistance Awareness among Smallholder Poultry Farmers in Murang’a County, Kenya.

## Data Availability

Raw data can be provided upon request to the corresponding author.

## Abbreviations

AMC: Amoxicillin-clavulanic acid
AMP: Ampicillin
AMR: Antimicrobial resistance
AST: Antimicrobial susceptibility testing
ATCC: American Type Culture Collection
BPW: Buffer peptone water
CAZ/CLA: Ceftazidime/Clavulanic acid
CAZ: Ceftazidime
CIP: Ciprofloxacin
CLSI: Clinical & Laboratory Standards Institute
CTX/CLA: Cefotaxime/clavulanic acid
CTX: Cefotaxime
ERY: Erythromycin
ESBL: Extended-spectrum β-lactamase
GDP: Gross Domestic Product (GDP)
GEN: Gentamicin
IMP: Imipenem
MALDI-TOF: Matrix-assisted laser desorption/ionization time-of-flight mass spectrometry
mCCDA: Modified charcoal-cefoperazone-deoxycholate agar
MDR: Multidrug-resistant
MHA: Muller Hinton agar
NAL: Nalidixic acid
STR: Streptomycin
STX: Trimethoprim/Sulfamethoxazole
TET: Tetracycline
WHO: World Health Organization
