# Supplementary material for: Antimicrobial use and resistance profiles of Escherichia coli and Campylobacter species from small-scale poultry farms in Central Kenya
Source: BMC Vet Res. 2026 Apr 27;22:342. doi: 10.1186/s12917-026-05505-9 (PMC13255289; doi:10.1186/s12917-026-05505-9)
Supplement: Supplementary file 1 — Supplementary Material 1. Supplementary File S1: Structured questionnaire used in the study - Assessment of Antimicrobial Use and Resistance Awareness among Smallholder Poultry Farmers in Murang’a County, Kenya. [file 12917_2026_5505_MOESM1_ESM.pdf]

Supplementary File S1: A Questionnaire to Assess Antimicrobial Use and Resistance Awareness among Smallholder Poultry Farmers in Murang'a County, Kenya

UNIVERSITY OF NAIROBI
FACULTY OF VETERINARY MEDICINE
DEPARTMENT OF VETERINARY PATHOLOGY, MICROBIOLOGY & PARASITOLOGY

Questionnaire: Assessment of Antimicrobial Use and Resistance Awareness among Smallholder Poultry Farmers in Murang'a County, Kenya

Project Title: Epidemiology of foodborne bacteria and associated antimicrobial resistance profiles of the isolates from chicken in Murang'a County, Kenya. A One Health approach

Consent Statement / Introduction

I, ________________________, am a Principal Investigator currently conducting a study on antimicrobial use and resistance profiles of foodborne pathogens in chicken and environmental samples in Murang'a County. You are kindly invited to participate in this survey by completing the following questionnaire. Your participation is voluntary, and your contribution is highly valued and appreciated. The information you provide will be treated with strict confidentiality and will be used solely for research purposes. Your identity will not be disclosed in any report or publication arising from this study.

By agreeing to participate, you give your consent to take part in this study. Thank you for your time and support.

Consent granted: **Yes/No.**

A. <u>Farm identification</u> **Date of Interview…….**

1. Sub County..................................Ward…………………………. Village........................
2. GPS reading: Eastings………………………. Southings…………………Altitude…….

B. <u>Farmer's Bio data</u>

1. Name of the farm owner/caretaker …………………………Mobile No…………………
2. Age of the farm owner/caretaker**-** [1]18-24 years [2] 25-31 years [3] 31-36 years [4] 37 years and above

3. Gender of farm owner/caretaker [1] Male [2] Female
4. Education level of the farm owner/caretaker
   [1] No Formal Education [2] Primary Level [3] Secondary Level [4] Tertiary Level
5. Level of income of farm owner/caretaker?..
6. What is your (respondent's) responsibility at the farm?
   [1] Owner [2] Worker [3] Other (Specify)………

C. **Information on poultry production**

7. What type of production do you practice?
   [1] Backyard Production [2] Free-range [3] Intensive production [4] Semi-intensive
8. What types/breeds of chicken do you have?
   [1] Kienyeji/local [2] Layers/ Broiler [3] Mixed breeds / Improved local breed
9. Flock size [1] ≥10 [2] 11-50 [3] 51-100 [4] ≤101
10. Other animals kept in the farm
    (i) Poultry [1] Yes [2] No
    (ii) Cattle [1] Yes [2] No
    (iii) Pigs [1] Yes [2] No
    (iv) Shoats [1] Yes [2] No
    (v) Donkey [1] Yes [2] No
    (vi) Others (Specify) ________________

D. **Farmer's knowledge and practice about antimicrobial use (AMU) and antimicrobial resistance (AMR)**

11. Have you ever heard about antibiotics? [1] Yes [2] No
12. Do you keep a record of using antimicrobials? [1] Always [2] Most frequently [3] Sometimes [4] Rarely [5] Never [6] Do not use antimicrobials
13. If yes above, please mention the names of common antibiotics you frequently use in poultry? (Provide photos of the commonly marketed antibiotics for use in Kenya, tick accordingly)
    Trade names……………………………………………………………………
    [1] Penicillin [2] Tetracycline/Doxycycline/Oxytetracycline [3] Streptomycin [4] Gentamycin
    [5] Cephalexine [6] Ceftriaxone [7] Ciprofloxacin [8] Cloxacillin [9] Cefixime
    [10] Sulphur-based drug (Sulfadimidine/Sulfadiazine/Sulphamethoxazole [11] Ampicillin [12], Amoxycillin
    [13] Ceftiofur [14] Lincomycin [15] Azithromycin [16] Amikacin [17] Others, Specify___
14. How often do you use antibiotics on the chicken? [1] Daily [2] Monthly or quarterly [3] Never
15. Why do you use antibiotics in your farm? [1] Whenever treating sick birds [2], Prevention of disease among healthy birds [3], to enhance/promote growth
16. Which diseases and/or conditions have you encountered on your farm in the last 6 months? If yes, which ones?
    ………………………………………………………………………………………
    ………………………………………………………………………………………
17. Antibiotics can be used to treat any kind of disease in animals. [1] Yes [2] No
18. Do you use the same antibiotics to prevent any specific disease? [1] Yes [2] No
19. Do you add antibiotics to the chicken feeds or water? [1] Yes [2] No
20. Number of antibiotics used at a time in your farm?
    [1] Single [2] Combined/Multiple [3] Both [1 & 2] [4] Do not use
21. What do you do with leftover antibiotics? [1] Keep for further use [2]. Throw in the garbage [3]. Give it to other farmers for use [4]. Bury in the ground/Burn
22. What does an antibiotic do? [1] Act against bacteria [2] Act against virus [3] Act against fungus/Others
    [4] Act against all of the above [5] Don't know

23. Have you ever heard about antimicrobial resistance? [1] Yes [2] No

24. What do you know about antimicrobial resistance?

 [1] It causes treatment failure [2]. It causes poor response to treatment [3]. Both [1 and 2]

 [4] Do not know [5] Others, Specify______________

25. Do you know incomplete antibiotic course may lead to antibiotic resistance?

 [1] Yes [2] No

26. Who recommends which antibiotics to be used in the chickens? [1] Veterinarian [2] Other farmers [3] Agrovet attendant [4] Representative of a pharmaceutical company [5] Veterinary Paraprofessional [6] Village Doctor/ Quack/CBHW [7] Self [8] Others, Specify___________________

27. Do you follow the exact prescription of the veterinarian when purchasing antibiotics?

 [No] [2] Always [2] Sometimes influenced by medicine seller/others

28. Do you follow a withdrawal period after treatment? [1] Yes [2] No

Thank you for your participation
